# A circulating microRNA signature as noninvasive diagnostic and prognostic biomarkers for nonalcoholic steatohepatitis

**DOI:** 10.1186/s12864-018-4575-3

**Published:** 2018-03-09

**Authors:** Jie Liu, Yue Xiao, Xikun Wu, Lichun Jiang, Shurong Yang, Zhiming Ding, Zhuo Fang, Haiqing Hua, Mark Stephen Kirby, Jianyong Shou

**Affiliations:** 1 Lilly China Research and Development Center, Shanghai, 201203 China; 2 Present Address: Fosun Kite Biotechnology, No. 222 Kangnan Road, Shanghai, 201210 China; 3 Present Address: Shanghai Ennova Biopharmaceuticals, 781 Cailun Road, Shanghai, 201203 China

**Keywords:** microRNA, NASH, Biomarker, ALT, miR-192, miR-21, miR-505

## Abstract

**Background:**

Noninvasive biomarkers are urgently needed for patients with nonalcoholic steatohepatitis (NASH) to assist in diagnosis, monitoring disease progression and assessing treatment response. Recently several exploratory studies showed that circulating level of microRNA is associated with NASH and correlated with disease severity. Although these data were encouraging, the application of circulating microRNA as biomarkers for patient screening and stratification need to be further assessed under well-controlled conditions.

**Results:**

The expression of circulating microRNAs were profiled in diet-induced NASH progression and regression models to assess the diagnostic and prognostic values and the translatability between preclinical mouse model and men. Since these mice had same genetic background and were housed in the same conditions, there were minimal confounding factors. Histopathological lesions were analyzed at distinct disease progression stages along with microRNA measurement which allows longitudinal assessment of microRNA as NASH biomarkers. Next, differentially expressed microRNAs were identified and validated in an independent cohorts of animals. Thirdly, these microRNAs were examined in a NASH regression model to assess whether they would respond to NASH treatment.

MicroRNA profiling in two independent cohorts of animals validated the up-regulation of 6 microRNAs (miR-122, miR-192, miR-21, miR-29a, miR-34a and miR-505) in NASH mice, which was designated as the circulating microRNA signature for NASH. The microRNA signature could accurately distinguish NASH mice from lean mice, and it responded to chow diet treatment in a NASH regression model. To further improve the performance of microRNA-based biomarker, a new composite biomarker was proposed, which consists of miR-192, miR-21, miR-505 and ALT. The new composite biomarker outperformed the microRNA signature in predicting NASH mice which had NAS > 3, and deserves further validations in large scale studies.

**Conclusion:**

The present study supported the translation of circulating microRNAs between preclinical models and humans in NASH pathogenesis and progression. The microRNA-based composite biomarker may be used for non-invasive diagnosis, clinical monitoring and assessing treatment response for NASH.

**Electronic supplementary material:**

The online version of this article (10.1186/s12864-018-4575-3) contains supplementary material, which is available to authorized users.

## Background

Nonalcoholic steatohepatitis (NASH) is histologically characterized by hepatic steatosis, ballooning, inflammation, and varying amount of pericellular fibrosis [1]. Patients are frequently either asymptomatic or have nonspecific symptoms, which impose a great obstacle for clinical diagnosis. Moreover, NASH patients are often associated with higher risk of developing both cardiovascular disease and type 2 diabetes, therefore early diagnosis and intervention would greatly benefit the patient by preventing the progression of major hepatic and extrahepatic manifestations [2, 3]. Currently, liver biopsy is still required in clinical diagnosis and assessment of the treatment response of NASH, despite its invasive nature, variability in sampling and potential complications [4]. Thus identification and development of circulating biomarkers for clinical applications are urgently needed.

MicroRNAs are small non-coding RNA molecules which are evolutionally conserved epigenetic regulators. A single microRNA could post-transcriptionally regulate hundreds of mRNAs through binding to the 3′-untranslated region and leading to its translational repression [5]. MicroRNAs are also present in extracellular spaces, and serve as intercellular messengers to affect many physiological processes [6, 7]. Circulating microRNA have emerged as attractive diagnostic tools for the assessment of the pathological state of their origin tissue [8–10]. Studies suggest that the circulating level of microRNA is associated with liver steatosis, inflammation, fibrosis and carcinogenesis [11, 12]. The performance of circulating microRNA alone or in combination with other serum biomarkers in NASH diagnosis has been explored in a number of clinical studies [13–15]. However, the significance of these studies is limited by the heterogeneity in disease status, patients’ genetic variations, and different analysis platforms [16]. For example, hepatic miR-34a is differentially expressed in NASH and physiologically associated with lipid metabolism, inflammation, and hormone signaling in NASH. However, the circulating level of miR-34a in NASH patients is not conclusive [17–19]. Given the important role of microRNA in NASH pathophysiology, the value and feasibility of circulating microRNA as non-invasive biomarkers need to be further investigated.

The present study aims at evaluating the diagnostic and prognostic value of circulating microRNA in a well-studied diet-induced NASH mouse model, in which potential genetic and environmental confounding effects are minimized. We started by reviewing the published data on circulating microRNA profile in NAFLD or NASH patients, and shortlisted 25 microRNAs which were reported to be differentially expressed [12, 15, 20]. Then we quantitatively measured the expression of these 25 microRNAs by qPCR in a time-dependent NASH progression model, to understand the association between circulating microRNA and disease evolution. A panel of differentially expressed microRNAs were identified and confirmed in an independent study, and was designated as circulating microRNA signature for NASH. The microRNA signature responded to NASH treatment and correlated well with disease regression. Based on these intriguing findings, the diagnostic power of circulating microRNA signature was assessed and a microRNA-based composite biomarker was proposed. Collectively, we established the translation of circulating microRNA between mice and men in NASH disease progression and regression. The new composite microRNA-based biomarker holds great promise in NASH diagnosis and prognosis, and warrants further evaluation in large scale translational studies.

## Methods

### Animal model generation

Male C57BL/6 mice were purchased from Nanjng Biomedical Research Institute. Mice were housed in a 12 h light-12 h dark cycle in a 21-23 °C facility. To induce NASH, mice at 4 weeks of age were fed ad libitum a high fat, high fructose and high cholesterol diet (3H diet, Research Diets Inc. D09100301). Control mice were fed a standard chow diet (LabDiet, 5C02C). Control and 3H diet mice were anesthetized in 5% isoflurane at varying time points following initiation of dietary intervention to assess liver histological lesions as described before [21]. Blood was collected by cardiac puncture, and plasma was separated by centrifugation at 10000 rpm for 10 min at 4 °C. Plasma samples were aliquoted and stored in − 80 °C freezer for biochemical assays and microRNA analysis. All procedures were performed according to protocols approved by the Institutional Animal Care and Use Committee (IACUC) of Lilly animal care and use ethical committees. A detailed description of diet treatment, histopathological analysis and biochemical assays were provided in an independent manuscript under preparation.

### Circulating microRNA detection

Total RNA was isolated from 50 μL of mouse plasma using miRNeasy serum/plasma kit (QIAGEN, 217184) following manufacturer’s instructions. MicroRNAs were measured by RT-qPCR method using customized IDEAL microRNA assay kits provided by MiRXES Singapore. Briefly, 5 μL of spike-in control microRNA was added into each RNA isolation reaction. The RNA quality and concentration were checked by NanoDrop 8000, and 135 ng total RNA were converted to cDNA using microRNA-specific primers in 40 μL reaction. The cDNA was further diluted 11 times, and 5 μL diluted cDNA were used in subsequent qPCR assays for each microRNA. The PCR reaction was run using ABI7900HT thermal cycler with the following settings: 95 °C-10 min; 40 °C-5 min; 95 °C-10 s, 60 °C-30 s, repeated for 40 cycles. The qPCR data were expressed as minus delta Ct using spike-in control as reference gene.

### Bioinformatics and statistical analysis

The qPCR data were expressed as minus delta Ct value using spike-in control as reference gene. The qPCR data were analyzed by hierarchical clustering, principle component analysis, random forest algorithms using MetaboAnalyst 3.0 web based data analysis suite without sample normalization, data transformation or data scaling [22]. Univariate/multivariate receiver operating characteristics (ROC) curve analysis were performed using MetaboAnalyst 3.0A as well, in which ALT concentrations were log transformed before analysis. All association studies were conducted using R-studio software.

## Results

### Circulating microRNA profiling revealed differential expression pattern between lean and NASH mice

**Fig. 1:**
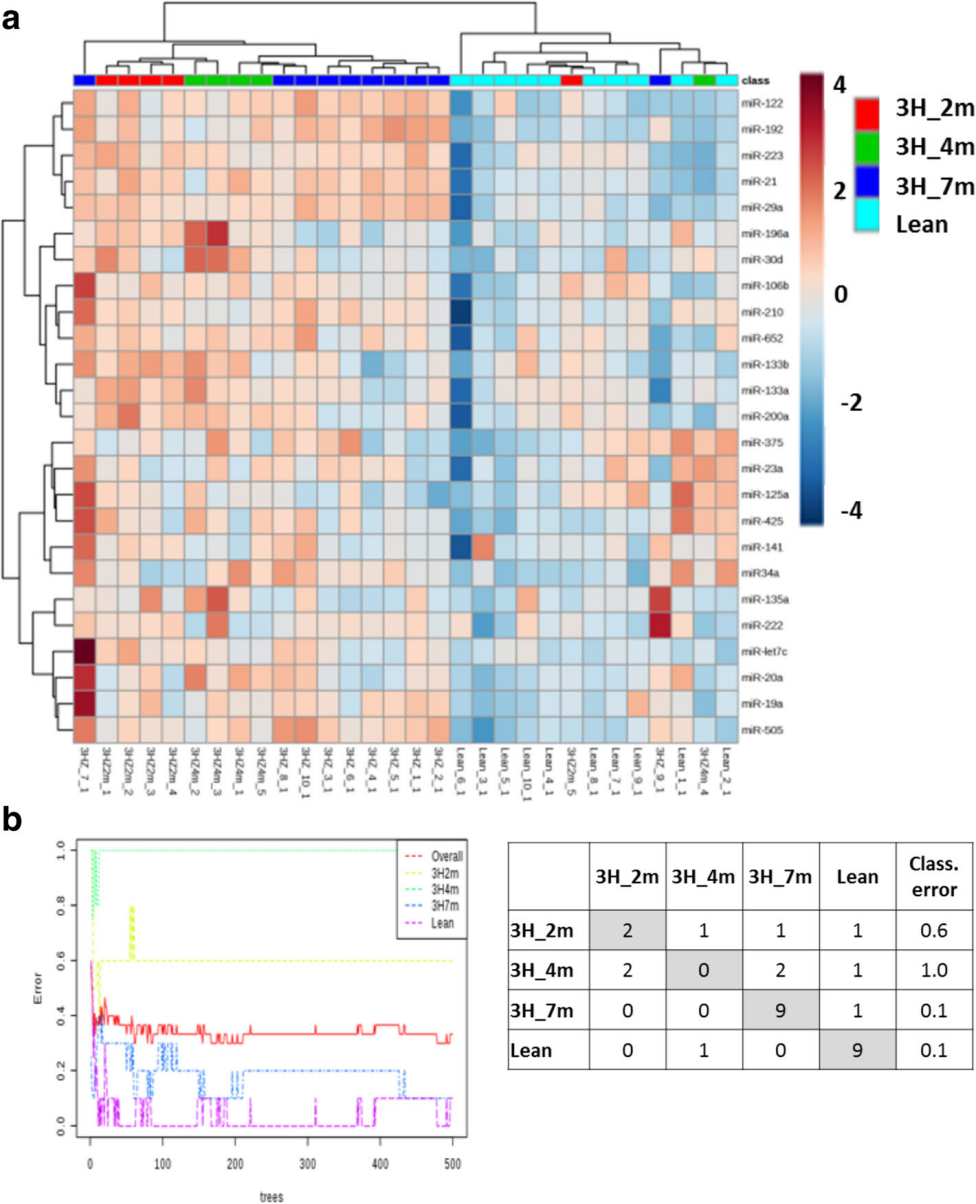
Circulating microRNA expression pattern in mice fed on 3H diet for varying time points compared with chow diet control mice. **a** Hierarchical clustering analysis revealed differential expression pattern between chow and 3H diet fed mice in circulating microRNA profile. The distance between features/samples were calculated by Euclidean method, and clustered using the Ward algorithm. **b** Random forest analysis on the microRNA expression data demonstrated a greater out-of-bag error in mice fed 3H diet for 2 and 4 months, suggesting heterogeneity in disease progression. Total number of trees were 500, and number of predictors were 7. Abbreviations: 3H, high fat, high fructose and high cholesterol; 3H_2m, 3H_4m, 3H_7m, mice on 3H diet for 2 months, 4 months, and 7 months respectively; lean, mice on standard chow diet; class. Error, classification error

The present study assessed the change in circulating microRNA expression in C57BL/6 mice fed on high fat, high cholesterol and high fructose diet (3H diet) in a time-course study. Mice on chow or 3H diet were terminated at 2 months (3H_2m, *n* = 5), 4 months (3H_4m, n = 5) or 7 months (3H_7m, *n* = 10; Lean, n = 10) for disease model characterization. Biochemical and histological studies showed that these mice developed obesity, dyslipidemia, hyperinsulinemia and liver injury in a time-dependent manner. Progressive hepatic steatosis and perisinusoidal fibrosis with concomitant elevations on ALT and AST were observed in 3H mice, which recapitulated the histological changes seen in human with NASH (The manuscript on detailed animal model characterization is in preparation.). Total 25 microRNAs were selected for profiling based on literature review of human studies (Complete list of microRNA was included in Additional file 1: Table S1). The microRNA was measured using ultrasensitive RT-qPCR assays which include three mature microRNA-specific primers in the detection system, and data were expressed as minus delta Ct with reference to spike-in control microRNA. Hierarchical clustering analysis revealed that there was distinct expression pattern of microRNAs between lean and 3H mice (Fig. 1a). Lean mice had low expression of microRNAs and were clustered together. Majority of 3H mice had high expression of microRNAs and were clustered together. In addition, a higher out of bag error rate was observed in mice on 3H diet for 2 and 4 months by random forest analysis, indicating that there is high degree of heterogeneity in NASH disease progression (Fig. 1b). It was also noted that the misclassified animal 3H_7m_9 had similar histopathological scores compared to other animals in 3H_7m group but with low expression of all microRNAs, which may imply for technical errors during microRNA isolation or detection. These data reveals distinct expression of circulating microRNAs between 3H diet treated and lean mice.

### A microRNA signature was identified and it accurately distinguished NASH mice from lean mice

**Fig. 2:**
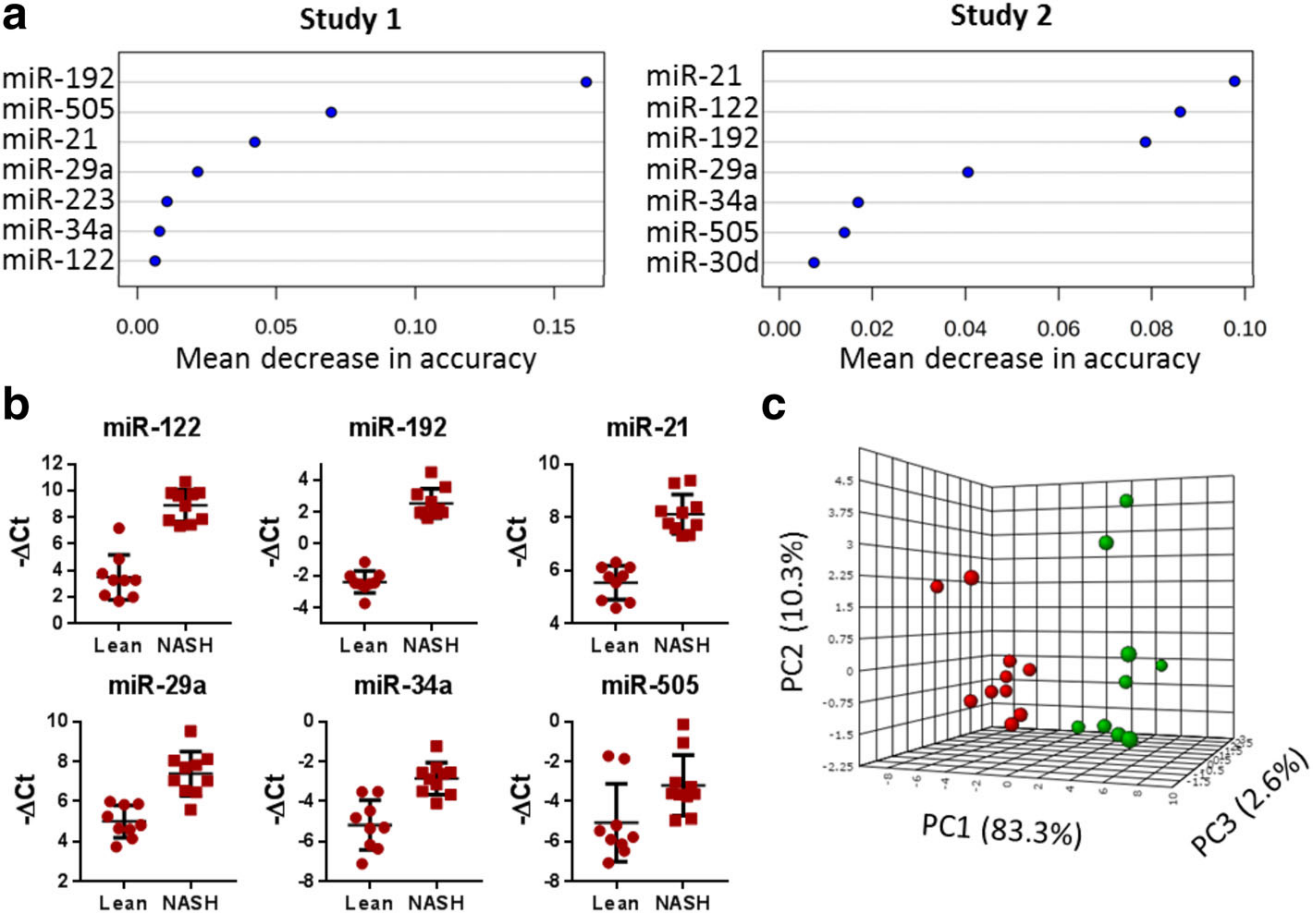
A panel of 6 microRNAs was designated as circulating microRNA signature for NASH. **a** Top seven microRNAs were listed and ranked by their classification accuracy in two independent studies by random forest analysis. **b** The expression level of the microRNA signature in lean and NASH mice in study 2. Data were expressed as -ΔCt (minus delta Ct) with reference to spike-in control microRNA. **c** Principle component analysis (PCA) of miR-192, miR-122, miR-21, miR-29a, miR-34a, and miR-505 in study 2. Red dots represented NASH mice (mice on 3H diet for 7 months), and green dots represented lean mice. Numbers in parenthesis mean the percentage of variance explained by each principle component

A second circulating microRNA profiling study was performed in an independent cohort to validate the data obtained from study one. Lean mice and NASH mice (on 3H diet for 7 months) were clearly separated on the heat map by hierarchical clustering analysis (Additional file 2: Figure S1). To further understand which microRNA(s) contribute most to the classification of NASH mice, the relative importance of individual microRNA was ranked by their contributions to classification accuracy in random forest analysis for both studies (Fig. 2a). The top six common microRNAs including miR-21, miR-122, miR-192, miR-29a, miR-34a and miR-505, were designated as the circulating microRNA signature for NASH (Fig. 2b). These microRNAs were significantly up-regulated in NASH mice in both studies, and their expression was correlated with the duration of 3H diet treatment (Additional file 3: Figure S2). Dimensional reduction by principle component analysis revealed that lean mice and NASH mice were distantly related based on their microRNA signature. As shown in Fig. 2c, the two groups of animals were clearly separated on the 3D-PCA plot, in which 96.2% of variance could be explained between NASH and lean mice. This set of data suggests that microRNA signature has excellent prediction power in distinguishing NASH mice, although the effect of sample homogeneity needs to be considered. Since these microRNAs were validated in human studies, our data established the translation of circulating microRNA between mice and men in NASH disease evolution and progression.

### The microRNA signature responded to chow diet treatment in NASH regression model

**Fig. 3:**
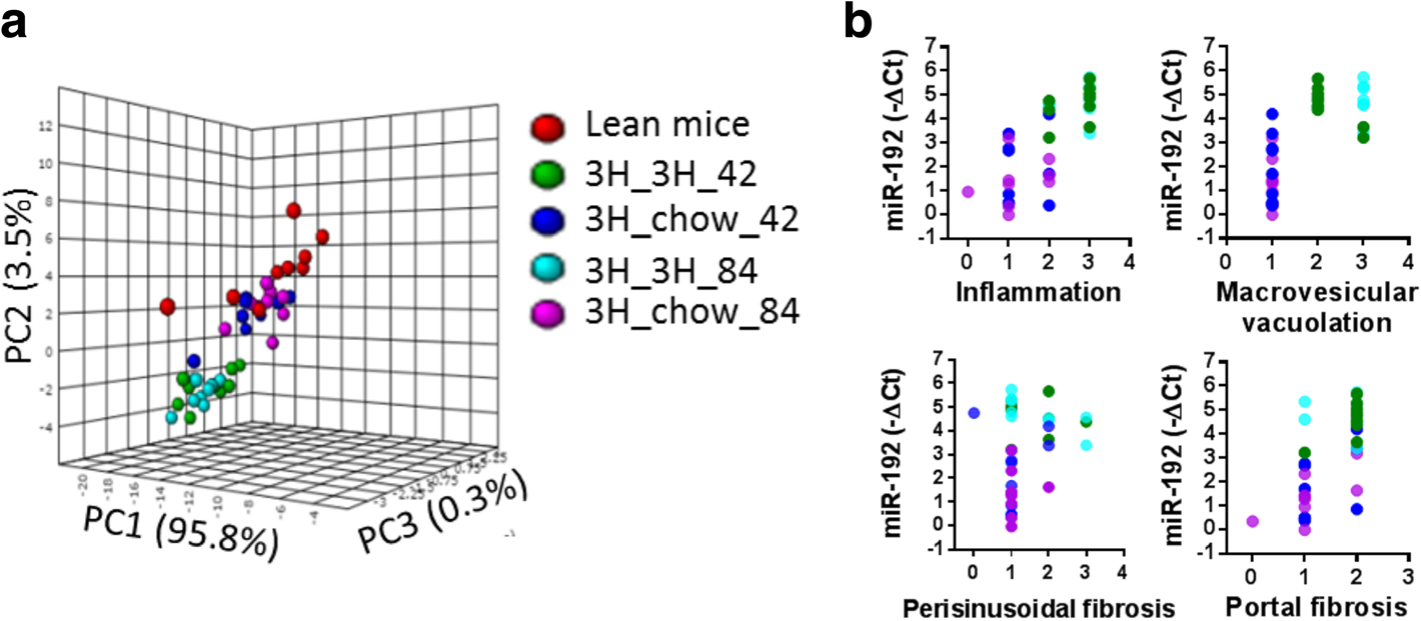
The microRNA signature responded to chow diet treatment in NASH mice. Mice were on 3H diet for 7 months for NASH development. Half of mice were switched to chow diet and were sacrificed on day 42 (3H_chow_42) or day 84 (3H_chow_84); half of mice were continued on 3H diet and were sacrificed on day 42 (3H_3H_42) or day 84 (3H_3H_84) as control. Lean mice were on standard chow diet and sacrificed together with the 3H_3H_84 group. **a** Principle component analysis of the microRNA signature in separating diet-switching animals (3H_chow_42, 3H_chow_84) from 3H mice (3H_3H_42, 3H_3H_84). **b** Scatter plots of miR-192 expression (reference to spike-in control) against histopathological scores: inflammation, macrovesicular vacuolation, perisinusoidal fibrosis and portal fibrosis

We then assessed whether the circulating microRNA signature could be reversed in a disease regression model. To test our hypothesis, the expression of microRNA signature was examined in NASH regression model, in which established NASH mice (with 7 months of 3H diet treatment) either continued on 3H diet or switched to chow diet for 42 and 84 days before termination for analysis. Three dimensional PCA plot revealed clear separation of lean mice from 3H mice groups (3H_3H_42 and 3H_3H_84). More importantly, 94.4% of mice switched to chow diet (3H_chow_42/84) were clustered with lean mice (Fig. 3a), indicating that the microRNA signature in diet-switching groups was normalized to an extent which is indistinguishable from that of lean mice. To further understand whether decrease in microRNA signature expression predicts disease regression, scatter plots on miR-192 expression against histological scores were generated (Fig. 3b). It was clear that diet-switching group animals had lower level of circulating miR-192 accompanied with improved liver histological lesions. Similar results were obtained for other signature microRNAs (data not shown). Altogether, these data independently validated the value of microRNA signature as diagnostic biomarkers for NASH, and further suggests the potential application in assessing treatment effects.

### Inclusion of ALT improved the diagnostic power of microRNA signature

**Fig. 4:**
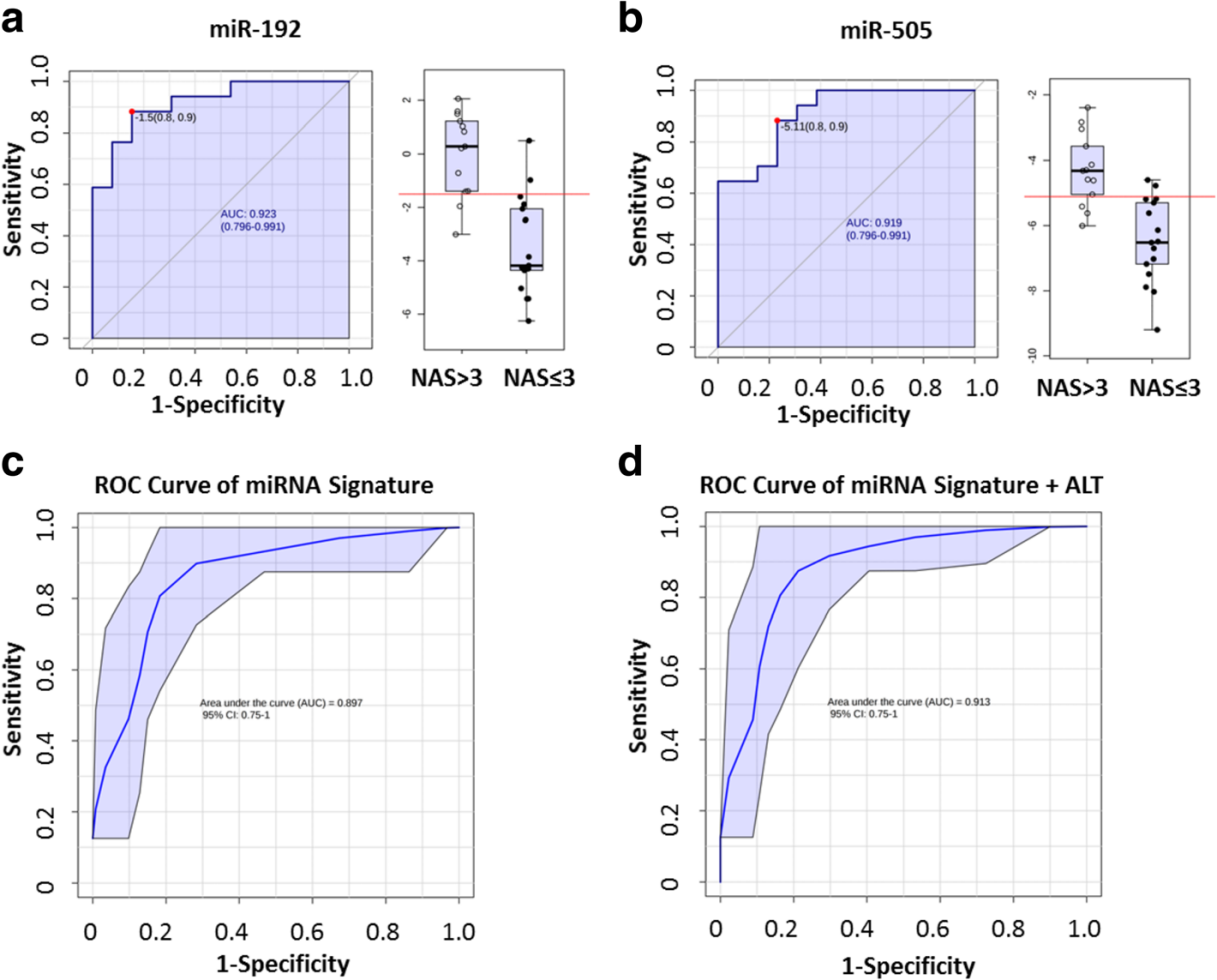
The diagnostic performance of microRNA signature in discriminating mice having NAS > 3 was improved by incorporating ALT. **a, b** Univariate ROC analysis of miR-192 and miR-505, which was created by plotting the true positive rate (sensitivity) against false positive rate (1-specificity). The right panel depicted the expression level of miR-192 and miR-505 in mice having NAS > 3 compared with mice having NAS ≤ 3. **c** ROC curves of the microRNA signature in predicting mice having NAS > 3 (AUC = 0.897, 95% confidence interval: 0.75 - 1) in study 1. The confusion matrix was depicted in the inset (numbers in rows are actual classification, numbers in columns are predicted classification). **d** ROC curves of the microRNA signature plus ALT in predicting mice having NAS > 3 (AUC = 0.913, 95% confidence interval: 0.75 - 1) in study 1. The confusion matrix was depicted in the inset. Abbreviations: NAS, NAFLD activity score; ROC, receiver operating characteristics; AUC, area under curve

Clinical diagnosis of NASH requires the histological examination of liver biopsies [23]. NAFLD activity score was developed by the pathology subcommittee of the NASH Clinical Research Network, and was validated by a group of expert hepatopathologists. In clinical practice, patients with NAS score of ≥5 strongly correlated with a diagnosis of “definite NASH”, whereas NAS ≤3 correlated with a diagnosis of “not NASH” [24]. We then assessed the performance of microRNA signature in discriminating mice which had NAS above 3 in study 1 because this cohort of mice had heterogeneous disease pathology. Univariate ROC curve analysis showed that miR-192 and miR-505 achieved the greatest AUROC of 0.923 and 0.919 respectively in discriminating mice had NAS > 3, while miR-122, miR-29a, miR-34a and miR-21 had an AUROC of 0.88, 0.84, 0.80 and 0.79 (Fig. 4a and b). To avoid potential overfitting of the data, multivariate ROC analysis was performed, and incorporation of all six microRNAs achieved the highest prediction power (AUROC = 0.897, 95% confidence interval: 0.75- 1) (Fig. 4b).

Liver transaminases (ALT and AST) have been widely used in clinical settings to predict the presence of liver diseases including NASH [25]. Elevations in circulating ALT level are indicative of hepatocyte damage, and ALT is an important component of several composite NASH scoring systems [26, 27]. As ALT and microRNAs are independent measurements of liver functions, we then sought to incorporate ALT into the microRNA signature to see if the performance could be further improved. As shown in Fig. 4c, the AUROC reached 0.913, and there was a reduction of 2 misclassified animals compared with microRNA signature model. These data demonstrated the feasibility of developing reliable circulating microRNA and ALT-based biomarkers to identify patients “at risk” for NASH thereby decreasing the need of liver biopsy during patient screening.

### Development of composite biomarkers for non-invasive diagnosis and prognosis of NASH

**Fig. 5:**
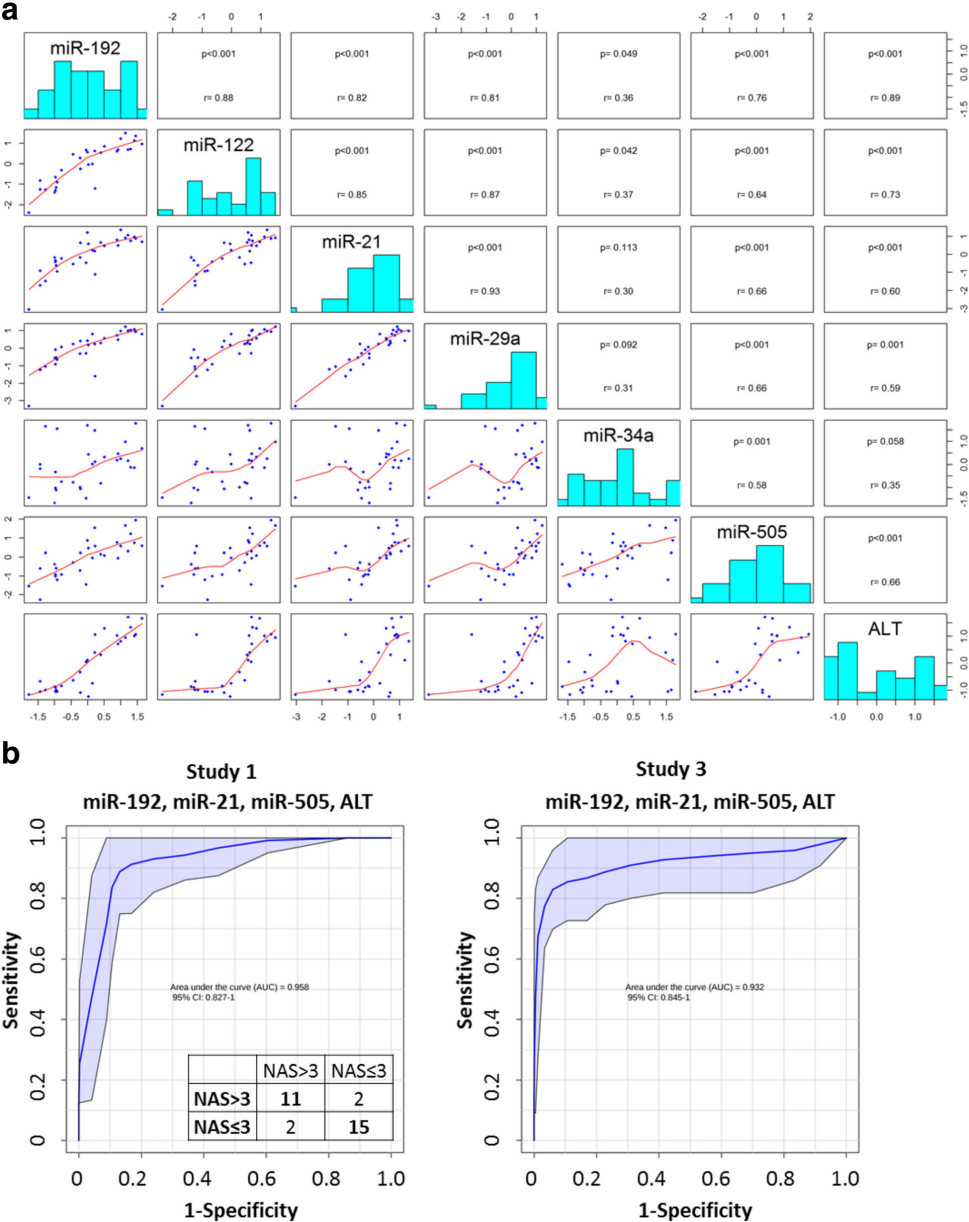
Development of non-invasive biomarker for NASH. **a** Associations between microRNAs and ALT was calculated. ALT concentration was log transformed before analysis. **b, c** ROC curves of the miR-192, miR-21, miR-505 and ALT in predicting mice having NAS > 3 in study 1 (AUC = 0.948, 95% confidence interval: 0.827 - 1) and study 3 (AUC = 0.931, 95% confidence interval: 0.845 - 1). The confusion matrix of study 1 was depicted in the inset

A robust non-invasive biomarker for NASH should be sensitive in disease discrimination, predictive of disease severity, and inexpensive for detection. The microRNA signature consists of 6 microRNAs, which may reduce the robustness and pose hurdles in future development. Thus, we sought to reduce the number of microRNAs by studying the associations among the microRNAs, and between microRNAs, and disease pathologies (Fig. 5a). MiR-192 and miR-122 fell into the same category, and both were implicated in liver injury and hepatocyte death [28, 29]. Since miR-192 had the highest association with other microRNAs and disease pathologies (NAS, *r* = 0.78), it was chosen to build the new composite biomarker. MiR-505 was selected because of its high correlations with disease pathologies (NAS, *r* = 0.77), but relatively low associations with other microRNAs. MiR-21 and miR-29a represented another cluster of microRNA, and both were functionally related to hepatic stellate cell activation and liver fibrosis [30–33]. We included miR-21 in the new biomarker because of its stronger human validation [13]. MiR-34a was weakly associated with disease pathologies or other microRNAs, thus it was omitted for further analysis. We then tested the performance of the new composite biomarker consisting of miR-192, miR-505, miR-21 and ALT in discriminating NASH animals (NAS > 3) from healthy mice (NAS ≤ 3) in study 1 and 3. As shown in Fig. 5b, the new biomarker outperformed the microRNA signature plus ALT model, and achieved AUROC of 0.958 with further reduction of one misclassified animal in study one. The new composite biomarker also accomplished excellent prediction accuracy in study three (AUROC of 0.932). Collectively, our data strongly validated the new composite biomarker in NASH diagnosis, and the performance of the new biomarker warrants further evaluation in large scale studies.

## Discussion

The present study evaluated the diagnostic and prognostic value of circulating microRNA in a well-studied diet-induced NASH mouse model. Expression of 25 selected microRNA was measured using a unique and ultrasensitive RT-qPCR based platform. A panel of 6 microRNA was validated to be differentially expressed in NASH mice and was correlated with disease severity. Based on these findings, a microRNA-based composite biomarker consisting of miR-192, miR-21, miR-505 and ALT was proposed, which demonstrated great performance in distinguishing between lean and NASH mice (NAS > 3). The composite biomarker mirrors the histological and molecular events in the liver, and potentially have both preclinical and clinical applications. In preclinical drug discovery, the composite biomarker could be used to evaluate disease progression and to assess treatment effects in mouse models. In clinical settings, the composite biomarker could be used for screening patients at risk for NASH, and/or used for monitoring treatment response, thus reducing the use of liver biopsies.

The microRNA based biomarkers demonstrated robust sensitivity in NASH diagnosis. In view of the biological function of each feature, the new composite biomarker could potentially reflect the status of the liver from the following perspectives: miR-192 and ALT serve as independent indicator of hepatocyte function, miR-21 suggests of stellate cell activation and liver fibrosis, miR-505 implies for pathological manifestations. MiR-192 regulates lipid synthesis in hepatocytes, and it was independently validated as biomarker for NASH by several groups using in vitro assays, animal models and clinical samples [12, 13, 34–36]. MiR-21 as biomarkers for hepatic fibrosis has been validated in both animal models and clinical samples [13, 37]. Studies have also shown that miR-21 regulates the peroxisome proliferator-activated receptor α (PPARα) activity and associates with stellate cell activation [31, 38]. Considering the association of miR-505 with various types of cancers, the biological function of miR-505 in NASH and liver carcinogenesis deserves further investigation [39]. Since these microRNAs originated from liver, their expression in NASH liver should also be considered. Previous studies have shown that liver miR-122 preferentially expressed in lipid-laden hepatocytes and was down-regulated in NASH patients [12]. Similarly, we observed slightly decrease in the liver miR-122 and miR-192 expression (not statistically significant) in NASH mice compared to lean mice. As miR-122 was very abundant in hepatocytes, a small number of hepatocyte degeneration may lead to significant release of miR-122 into circulation. Notably, we detected significant up-regulation of liver miR-21, miR-34a and miR-505 in NASH mice compared to lean mice (data was not shown). Since these microRNAs have been implicated in hepatic lipid homeostasis, inflammation, stellate cell activation and carcinogenesis [9, 37, 40, 41]; our results strongly support that the circulating level of microRNA could reflect the status of the liver, and hence could be employed as non-invasive diagnostic tools for NASH.

MicroRNAs are epigenetic regulators, which post-transcriptionally modulate hundreds of target genes. The experimental validated target genes of the microRNA signature were identified by miRTarBase [42]. Ingenuity Pathway Analysis of the target genes reveal that the top network was endocrine disorders, gastrointestinal disease, and metabolic disorders. It also suggests that EGFR signaling pathway may be a potential target for liver regeneration following chronic liver damage in NASH. Indeed, Erlotinib treatment in carbon tetrachloride-induced mouse fibrosis model attenuated liver fibrosis and the development of hepatocellular carcinoma [43]. These findings suggest that functional microRNA – mRNA network analysis may represent a novel approach in the discovery of new drug target for the treatment of NASH.

Considering future directions, we think that development of reliable internal normalization strategy is critical. We used spike-in microRNA control as internal reference, however, it might not be ideal for clinical use as the relative microRNA expression might be influenced by batch-to-batch variance resulting from RNA isolation. Therefore we propose that identification of house-keeping microRNAs or generation of statistical internal normalization algorithms are urgently needed for the absolute quantification of circulating microRNAs in NASH patients for accurate diagnosis and staging of the disease.

MicroRNAs in serum or plasma are relatively stable as they are associated with membrane-bound vesicles or proteins. The ease of sample handling and the availability of inexpensive qPCR-assays make them ideal for use in clinic settings for patient screening and stratification. Although our data are promising, the main limitation was that the current study focused on a selected panel of microRNAs. Future effort should be devoted to global screening studies to identify microRNAs with the best diagnostic and prognostic performance specific for NASH.

## Conclusion

The present circulating microRNA profiling studies assessed the expression of 25 selected microRNAs in a diet-induced NASH mouse model. Our data validated the differential expression of a panel of microRNAs in NASH models, and supported the translation between preclinical and clinical studies. The circulating microRNA based biomarker proposed here holds great promise for clinical application, and deserves further evaluation in clinical studies.

## Additional files

Additional file 1: **Table S1.** Complete list of microRNAs analyzed. (DOCX 17 kb)

Additional file 2: **Figure S1.** Hierarchical clustering analysis revealed differential expression of circulating microRNAs between lean and 3H mice in an independent study (study 2). (DOCX 347 kb)

Additional file 3: **Figure S2.** Time-dependent expression of miR-122, miR-192, miR-21, miR-29a, miR-34a, and miR-505 in study 1. Data were expressed as minus delta Ct with reference to spike-in control miRNA. (DOCX 149 kb)

## Abbreviations

ALT: Alanine transaminase
AST: Asparte transaminase
AUROC: Area under ROC
MicroRNA: microRNA
NAFLD: Nonalcoholic fatty liver disease
NAS: NAFLD Activity score
NASH: Nonalcoholic steatohepatitis
PCA: Principle component analysis
qPCR: Quantitative polymerase chain reaction
ROC: Receiver operating characteristic
