# Supplementary material for: A circulating microRNA signature as noninvasive diagnostic and prognostic biomarkers for nonalcoholic steatohepatitis
Source: BMC Genomics. 2018 Mar 9;19:188. doi: 10.1186/s12864-018-4575-3 (PMC5845150; doi:10.1186/s12864-018-4575-3)
Supplement: Supplementary file 1 — Table S1. Complete list of microRNAs analyzed. (DOCX 17 kb) [file 12864_2018_4575_MOESM1_ESM.docx]

**Additional file 1**: Table S1. Complete list of microRNAs analyzed

| **list of miRNA profiled** | |
| --- | --- |
| 1 | has-miR-133a-3p |
| 2 | has-miR-135a-5p |
| 3 | has-miR-125a-5p |
| 4 | has-miR-106b-5p |
| 5 | has-miR-375 |
| 6 | has-miR-23a-3p |
| 7 | has-miR-133b |
| 8 | has-miR-425-3p |
| 9 | has-miR-200a-5p |
| 10 | has-let-7c-5p |
| 11 | has-miR-652-3p |
| 12 | has-miR-196a-5p |
| 13 | has-miR-30d-5p |
| 14 | has-miR-141-3p |
| 15 | has-miR-210-3p |
| 16 | has-miR-222-3p |
| 17 | has-miR-192-5p |
| 18 | has-miR-19a-3p |
| 19 | has-miR-20a-5p |
| 20 | has-miR-21-5p |
| 21 | has-miR-223-3p |
| 22 | has-miR-29a-3p |
| 23 | mmu-miR-34a-3p |
| 24 | mmu-miR-505-3p |
| 25 | miRNA Spike-in 1 |
