# Supplementary material for: A circulating microRNA signature as noninvasive diagnostic and prognostic biomarkers for nonalcoholic steatohepatitis
Source: BMC Genomics. 2018 Mar 9;19:188. doi: 10.1186/s12864-018-4575-3 (PMC5845150; doi:10.1186/s12864-018-4575-3)

**Additional file 2**: Figure S1. Hierarchical clustering analysis revealed differential expression of circulating microRNAs between lean and 3H mice in an independent study (study 2).


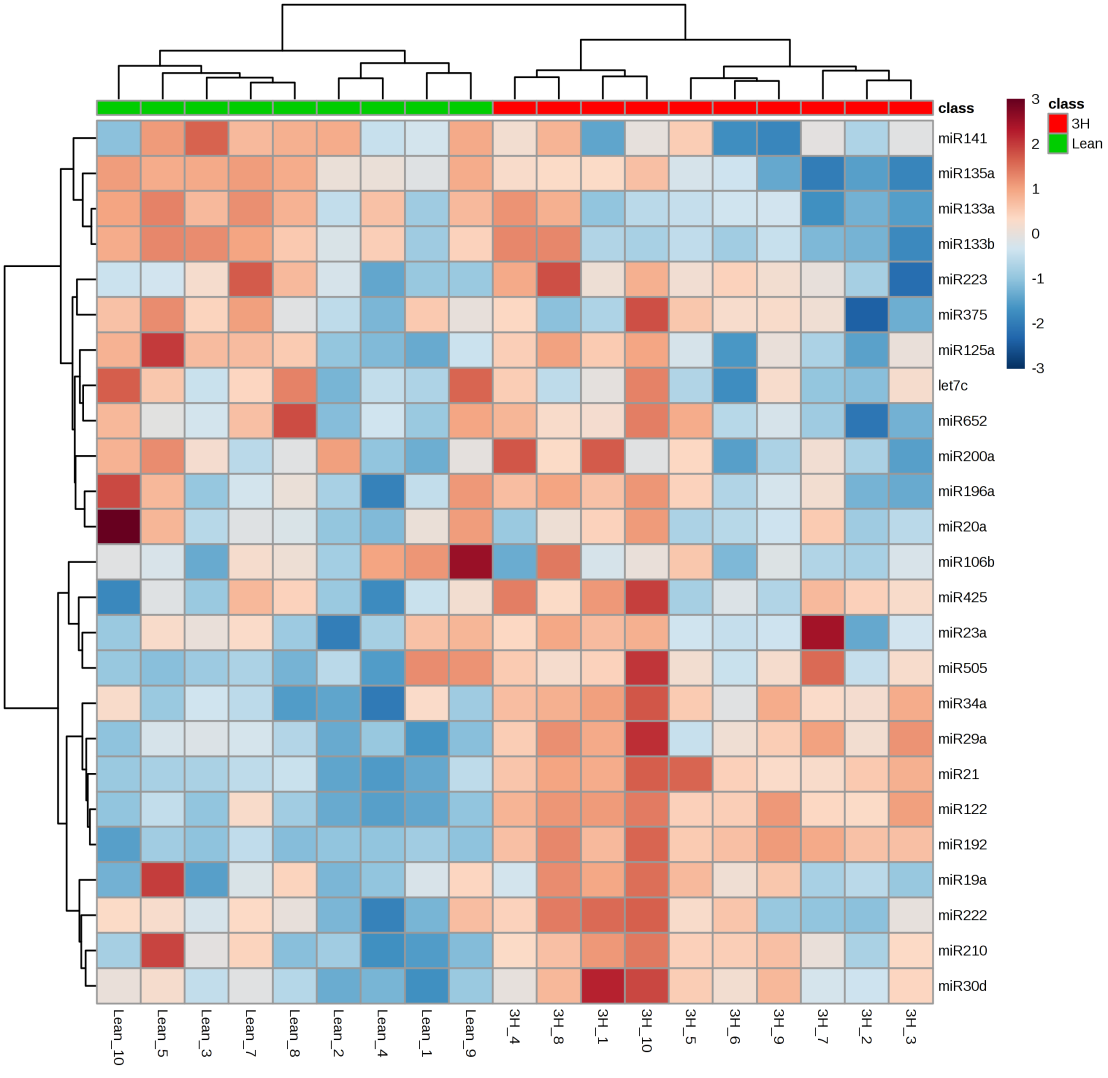

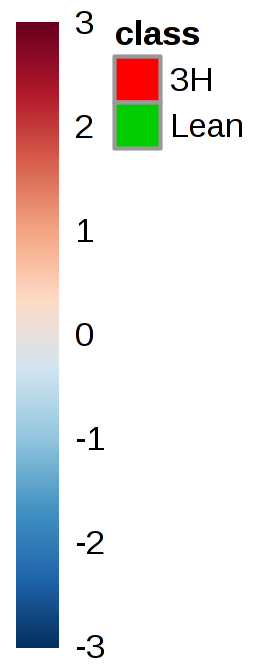

Supplement: Supplementary file 2 — Figure S1. Hierarchical clustering analysis revealed differential expression of circulating microRNAs between lean and 3H mice in an independent study (study 2). (DOCX 347 kb) [file 12864_2018_4575_MOESM2_ESM.docx]
