# Supplementary material for: A circulating microRNA signature as noninvasive diagnostic and prognostic biomarkers for nonalcoholic steatohepatitis
Source: BMC Genomics. 2018 Mar 9;19:188. doi: 10.1186/s12864-018-4575-3 (PMC5845150; doi:10.1186/s12864-018-4575-3)
Supplement: Supplementary file 3 — Figure S2. Time-dependent expression of miR-122, miR-192, miR-21, miR-29a, miR-34a, and miR-505 in study 1. Data were expressed as minus delta Ct with reference to spike-in control miRNA. (DOCX 149 kb) [file 12864_2018_4575_MOESM3_ESM.docx]

**Additional file 3**: Figure S2. Time-dependent expression of miR-122, miR-192, miR-21, miR-29a, miR-34a, and miR-505 in study 1. Data were expressed as minus delta Ct with reference to spike-in control miRNA.

Duration on 3H diet (Months)
